# Antifungal, Antibacterial, and Antioxidant Activities of *Acacia Saligna* (Labill.) H. L. Wendl. Flower Extract: HPLC Analysis of Phenolic and Flavonoid Compounds

**DOI:** 10.3390/molecules24040700

**Published:** 2019-02-15

**Authors:** Asma A. Al-Huqail, Said I. Behiry, Mohamed Z. M. Salem, Hayssam M. Ali, Manzer H. Siddiqui, Abdelfattah Z. M. Salem

**Affiliations:** 1 Chair of Climate Change, Environmental Development and Vegetation Cover, Department of Botany and Microbiology, College of Science, King Saud University, Riyadh 11451, Saudi Arabia; aalhuquail@ksu.edu.sa (A.A.A.-H.); hayhassan@ksu.edu.sa (H.M.A.); mhsiddiqui@ksu.edu.sa (M.H.S.); 2 Agricultural Botany Department, Faculty of Agriculture (Saba Basha), Alexandria University, Alexandria 21531, Egypt; behiry_2006@yahoo.com; 3 Forestry and Wood Technology Department, Faculty of Agriculture (El-Shatby), Alexandria University, Alexandria 21545, Egypt; 4 Timber Trees Research Department, Sabahia Horticulture Research Station, Horticulture Research Institute, Agriculture Research Center, Alexandria 21526, Egypt; 5 Facultad de Medicina Veterinaria y Zootecnia, Universidad Autónoma del Estado de México, 50000 Estado de México, Mexico

**Keywords:** *acacia saligna*, antibacterial activity, antifungal activity, antioxidant activity, flowers, wood-treated extract

## Abstract

In this study, for the environmental development, the antifungal, antibacterial, and antioxidant activities of a water extract of flowers from *Acacia saligna* (Labill.) H. L. Wendl. were evaluated. The extract concentrations were prepared by dissolving them in 10% DMSO. Wood samples of *Melia azedarach* were treated with water extract, and the antifungal activity was examined at concentrations of 0%, 1%, 2%, and 3% against three mold fungi; *Fusarium culmorum* MH352452, *Rhizoctonia solani* MH352450, and *Penicillium chrysogenum* MH352451 that cause root rot, cankers, and green fruit rot, respectively, isolated from infected *Citrus sinensis* L. Antibacterial evaluation of the extract was assayed against four phytopathogenic bacteria, including *Agrobacterium tumefaciens, Enterobacter cloacae, Erwinia amylovora,* and *Pectobacterium carotovorum subsp. carotovorum*, using the micro-dilution method to determine the minimum inhibitory concentrations (MICs). Further, the antioxidant capacity of the water extract was measured via 2,2′-diphenylpicrylhydrazyl (DPPH). Phenolic and flavonoid compounds in the water extract were analyzed using HPLC: benzoic acid, caffeine, and *o*-coumaric acid were the most abundant phenolic compounds; while the flavonoid compounds naringenin, quercetin, and kaempferol were identified compared with the standard flavonoid compounds. The antioxidant activity of the water extract in terms of IC_50_ was considered weak (463.71 μg/mL) compared to the standard used, butylated hydroxytoluene (BHT) (6.26 μg/mL). The MIC values were 200, 300, 300, and 100 µg/mL against the growth of *A. tumefaciens*, *E. cloacae*, *E. amylovora*, and *P. carotovorum subsp. carotovorum*, respectively, which were lower than the positive control used (Tobramycin 10 μg/disc). By increasing the extract concentration, the percentage inhibition of fungal mycelial was significantly increased compared to the control treatment, especially against *P. chrysogenum*, suggesting that the use of *A. saligna* flower extract as an environmentally friendly wood bio-preservative inhibited the growth of molds that cause discoloration of wood and wood products.

## 1. Introduction

Wide global issue of foodborne diseases influenced significantly on environmental development and health. Consumers demand growing day by day for natural preservatives as alternatives to solve the bad reputation of toxic chemical compounds. The Plant extracts had antimicrobial compounds must be thoroughly characterized for their endo potential to serve as biocontrol or biopreservative agents. Comprehensive papers focused on plant extracts as antimicrobial agents for use in preservation and control foodborne pathogens in foods. Medicinal and aromatic plants rich in phytochemical compounds such as polyphenols, flavonoids, saponins, alkaloids, and others in their different parts (leaves, bark, flowers, seeds, wood, and branches) have broad applications as antioxidants and antimicrobials and are known for their pharmaceutical and biopesticide properties [1,2,3,4,5,6]. Several mold species, such as *Fusarium*, *Paecilomyces*, *Rhizoctonia*, *Penicillium*, *Aspergillus*, *Alternaria*, and *Trichoderma*, can colonize and cause pigmentation in, colored spores on, and the discoloration of different wood and wood-based products in humid conditions [7,8,9,10]. Molds produce hydrolyzing enzymes that hydrolyze cellulose into glucose [11], xylanase enzymes [12], and *β*-xylosidases that hydrolyze hemicelluloses [13]. In-service wood can be fortified against mold growth by using natural products as a surface experiment application [14,15,16].

*Fusarium culmorum* is a ubiquitous soil-borne fungus able to cause root rot on citrus specimens, particularly oranges and lemons [17]. However, the most frequently isolated fungi from the rotted roots of lemon transplants were *F. oxysporum*, *F. solani,* and *R. solani*, showing root rot and wilt disease complexes. The average percentage of root rot/wilt incidence in surveyed districts was 34.0% in lemon [18]. Meanwhile, the *Penicillium spp.*, considered the most important postharvest fungal pathogen, reported on citrus and stone fruits were *P. chrysogenum*, *P. crustosum*, and *P. expansum* [19,20,21,22], and accounted for up to 90% of total losses [23,24]. *Fusarium* species and their fumonisin mycotoxins are toxic, causing maize ear rot disease and contaminating maize grains, leading to major problems in pre- and post-harvest losses [25,26]. Additionally, Panama wilt disease is caused by *F. oxysporum* in bananas (*Musa paradisiaca*) [27].

Phytopathogenic bacteria Agrobacterium tumefaciens, *Bacillus pumilus*, *Dickeya solani*, *Enterobacter cloacae*, *Ralstonia solanacearum*, and *Pectobacterium carotovorum subsp. carotovorum* are causal agents of different infectious plant symptoms, such as blackleg, brown or soft rot on potato tuber and stems, and tumors on olive and other ornamental plants [28,29,30,31]. Therefore, several studies have been carried out to study the effects of natural extracts on these bacteria and have shown a range of weak to strong activity [22,24,29,30,31].

A wide array of polymerase chain reaction (PCR) and real-time PCR tools, as well as complementary methods, have been developed for the detection and quantification of *F. culmorum*, *R. solani*, and *P. chrysogenum* in isolated pure cultures and in naturally infected plant tissue [32,33,34,35]. Presently, a great challenge in agriculture is the control of plant diseases caused by phytopathogenic bacteria and fungi. The emergence of antifungal/antibacterial-resistant strains is increasing, emphasizing the urgent need for the development of novel antifungal agents with properties and mechanisms of action different from the existing ones [32].

Currently, the fungicides/bactericides used are costly and environmentally toxic [36]. Also, phytopathogenic fungi and bacteria have developed resistance to most of the conventional pesticides and antibiotics [37,38]. Therefore, a search for new sources of biocides is needed from tropical and subtropical plants rich in phytochemicals that could be used as defense compounds for the protection of crop plants [39,40].

*Acacia saligna* (Labill.) H. L. Wendl. (*Acacia cyanophylla* Lindl.), native to Western Australia and belonging to the family Fabaceae, has been planted in Egypt and other Mediterranean countries in Africa, become an invasive species, and is considered a fast-growing tree [41,42]. Different Acacia plants produce allelopathic materials as bioactive compounds [43,44]; where phenolics, tannins, flavonoids, phenols, and proanthocyanidins are the most common compounds identified in various parts of the *Acacia* species [45,46,47,48,49]. The methanol extract of flowers and leaves was observed to have allelopathic effects greater than those of an aqueous extract on the germination percentage of *Hordeum murinum* [50], which was caused by a variety of active components [51]. The ethyl acetate extract of *Acacia saligna* leaves was more effective against *Staphylococcus aureus, S. pyogenes, Bacillus cereus, B. subtilis,* and *Candida albicans* than methanolic and water extracts [52].

The aim of this study was to evaluate the bioactivity of a water extract from flowers of *Acacia saligna* in terms of the fungal resistance of wood treated with the water extract, antibacterial activity against some phytopathogenic bacteria, and antioxidation properties. Further, the main phenolic and flavonoid compounds in the water extract were analyzed using an HPLC method.

## 2. Results

### 2.1. Isolated Fungi

The isolation trails and the ITS sequences revealed that the three fungal isolates were *Fusarium culmorum*, *Rhizoctonia solani*, and *Penicillium chrysogenum*. GenBank accession numbers of the isolated fungi are listed in Table 1.

### 2.2. Antifungal Activity of Wood Treated with Water Extract

**Figure 1:**
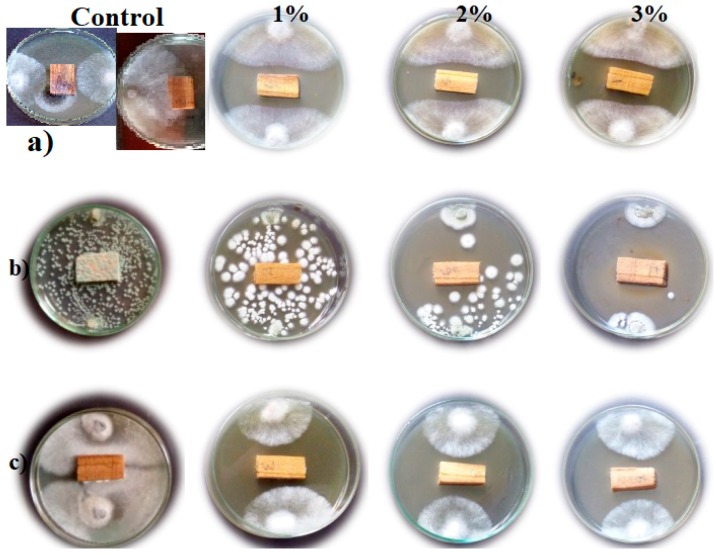
Wood treated with water extracts of *A. saligna* flowers and exposed to the growth of three fungi: (**a**) *Rhizoctonia solani*; (**b**) *Penicillium chrysogenum*; and (**c**) *Fusarium culmorum*.

Figure 1 shows that wood samples treated with water extracts of different concentrations (1, 2, and 3%) of *A. saligna* flower presented different degrees of inhibitions to fungal growth compared to the control treatment (wood samples treated with 10% DMSO). The water extract of flowers at 3% exhibited the highest inhibition percentage of mycelial growth of *F. culmorum*, *P. chrysogenum*, and *R. solani*, with values of 38.51%, 65.92%, and 41.48%, respectively, compared to the control. It can be seen from Table 2 that there is no significant difference among the three concentrations (1, 2, and 3%) of water extracts for the inhibition percentage of *R. solani*. However, with an increasing concentration, the percentage inhibition of fungal mycelia of *P. chrysogenum* was significantly increased. Furthermore, there is no significant difference between the concentrations 1% and 2% for the inhibition percentage of *F. culmorum*, but the concentration 3% showed the highest inhibition percentage with a significant value against the same fungus.

### 2.3. Antibacterial Activity

Table 3 presents the antibacterial activity of the water extracts, where the MIC values in µg/mL of 200 (*A. tumefaciens*), 300 (*E. cloacae*), 300 (*E. amylovora*), and 100 (*P. carotovorum subsp. carotovorum*) were observed and all the values were lower than those reported from the positive control used (Tobramycin 10 μg/disc).

### 2.4. Phytochemical Constituents and DPPH Activity of Extract

**Figure 2:**
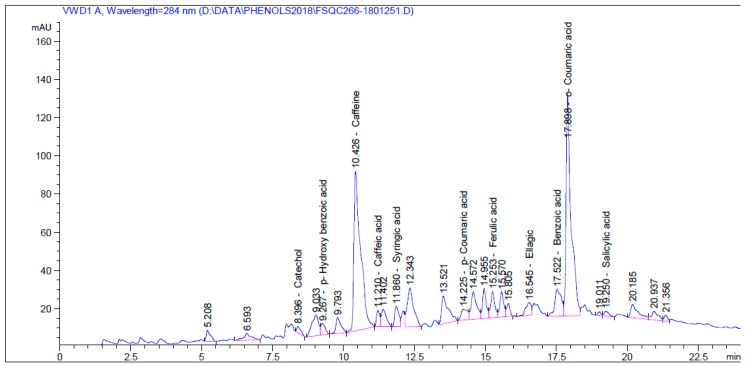
HPLC chromatogram of phenolic compounds identified in water extract of *A. saligna* flowers.

**Figure 3:**
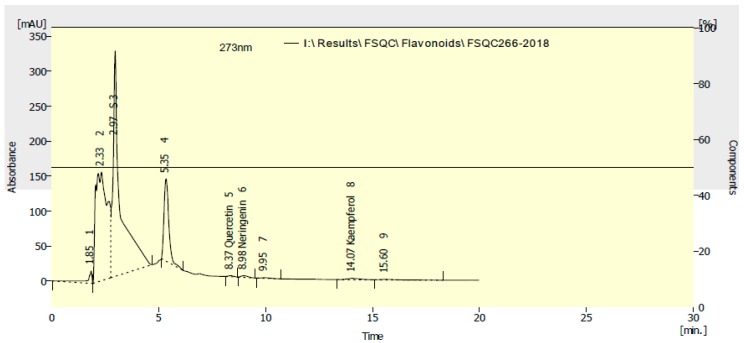
HPLC chromatogram of flavonoid compounds identified in water extract of *A. saligna* flowers.

The HPLC chromatograms for the identified phenolic and flavonoid compounds are shown in Figure 2 and Figure 3, respectively. Table 4 presents the phenolic and flavonoid compounds identified in the water extract of *A. saligna* flowers. The most abundant phenolic compounds were benzoic acid (161.68 mg/100 g), caffeine (100.11 mg/100 g), *o*-coumaric acid (42.09 mg/100 g), *p*-hydroxy benzoic acid (14.13 mg/100 g), and ellagic acid (12.17 mg/100 g); while the identified flavonoid compounds were naringenin (145.03 mg/100 g), quercetin (111.96 mg/100 g), and kaempferol (44.49 mg/100 g).

**Figure 4:**
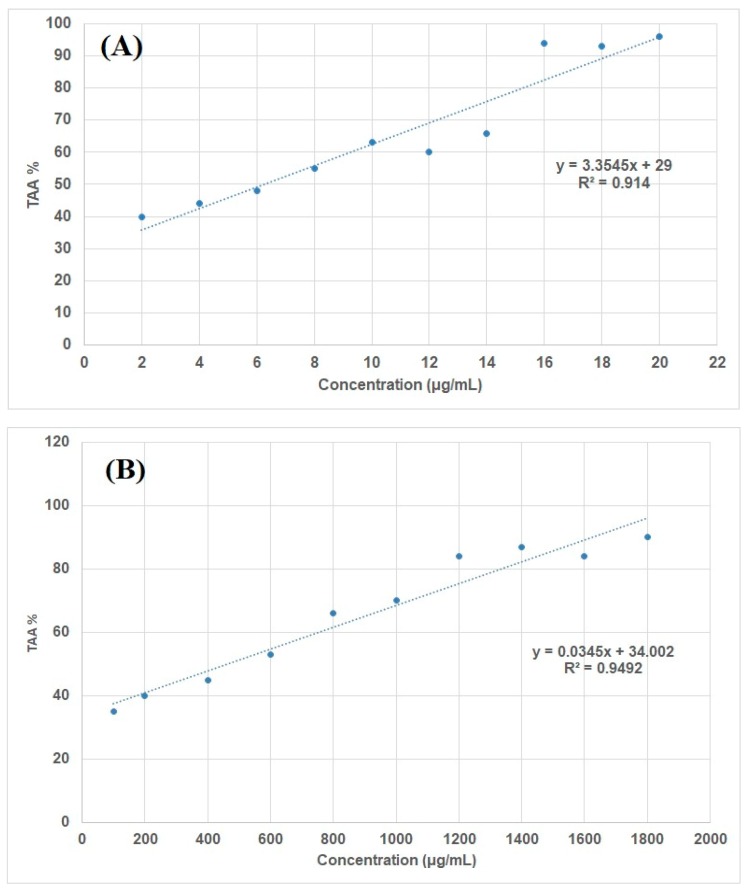
TAA % curve of BHT (**A**) and water extract from *A. saligna* flowers (**B**).

A spectrophotometric DPPH assay was used to evaluate the antioxidant properties of the flower water extract. Results of the antioxidant activity of the water extract from *A. saligna* flowers showed that with increasing concentration, the total antioxidant activity (TAA%) increased (Figure 4). The results obtained show that the total flower extract presents a weak scavenging capacity. The measured antioxidant activity in terms of the IC_50_ value (the concentration of the extract able to scavenge 50% of the DPPH free radical) was 463.71 μg/mL, which was lower than the value reported with BHT (6.26 μg/mL).

## 3. Discussion

Bioactive compounds extracted from plant materials are recognized to be the first step for the utilization of phytochemicals in dietary supplement preparation, as well as of food ingredients, pharmaceutical products, and antimicrobial agents [2]. Several studies have examined the antimicrobial activity of water extracts from *Acacia* species. The aqueous extract of *A. cyanophylla* leaves showed different levels of activity against the growth of *Staphylococcus aureus*, *Bacillus subtilis*, *Pseudomonas aeruginosa*, *Xanthomonas*, *Escherichia coli*, and *Citrobacter* [53]. The extract obtained with *A. saligna* leaves was effective against the tested bacteria *B. subtilis*, *E. coli*, *Klebsiella pneumoniae*, *P. aeruginosa*, *S. aureus*, and *Micrococcus luteus* (Gumgumjee and Hajar, 2015). The ethanol extract of *A. saligna* showed good activity against *A. niger*, *A. fumigatus*, *A. flavus*, and *C. albicans* at a concentration of 200 mg/mL [54]. To the best our knowledge, no studies have been carried out to study the effects of flower extracts against the phytopathogenic bacteria. Other studies showed that alkaloidal extracts from *Conocarpus lancifolius* leaves had MICs value of 20–50 μg/mL and >200 μg/mL against the growth of *A. tumefaciens* and *E. amylovora*, respectively [55]; while it was 125, 250, 125, and 16 μg/mL against *P. carotovorum subsp. carotovorum* when treated with acetone extract of *Callistemon viminalis* flower, *n*-butanol extract of *C. viminalis* flower, essential oil from the aerial parts of *Conyza dioscoridis*, and *n*-butanol extract from the bark of *Eucalyptus camaldulensis*, respectively; and against *A. tumefaciens* with values of 16, 250, >4000, and 250 μg/mL with the same extracts [3]. The leaf ethanol extract of *A. cyanophylla* showed activity against *Aspergillus niger*, *A. fumigatus*, *A. flavus*, and *Candida albicans*, where *A. fumigatus* was the most susceptible fungi and *A. niger* the most resistant [56].

Various *Acacia* species have condensed and hydrolysable tannins, as well as flavonoids [57,58]; which exhibit various bioactivities, such as antioxidant, anti-inflammatory, and antimicrobial properties [59]. *A. saligna* extracts showed steroidal saponins and biflavonoid glycosides [60], with high antimicrobial activity. Catechin, 7-*O*-galloylcatechin, myricetin-3-*O*-*α*-l-arabinopyranoside, quercetin-3-*O*-*β*-*d*-glucopyranoside, quercetin-3-*O*-*α*-*l*-arabinopyranoside, apigenin-7-*O*-*β*-*d*-glucopyranoside, and luteolin-7-*O*-*β*-*d*-glucopyranoside were isolated from *A. saligna* leaves [52]. Several flavonoid compounds (astragalin, myricitrin, and quercitrin) have been isolated from the leaves of *A. saligna* [60]. Myricetin 3-*O*-glucoside has also been found in *A. saligna* [61].

Phenolic and flavonoid compounds extracted from different plant materials have shown potential antioxidant properties, i.e., flavonoids from *Larix decidua* [62] and *Abies spectabilis* [63] bark extracts. The catechol group presented in the quercetin derivative is known to strongly increase activity in the DPPH assay [64]. On the other hand, moderate and lower antioxidant activity than standard antioxidants (butylated hydroxyanisole (BHA), butylated hydroxytoluene (BHT), and ascorbic acid), was found with ethanolic extracts of *Salvia microstegia*, *S. brachyantha,* and *S aethiopis*, while the main phenolic and flavonoid compounds were kaempferol, rosmarinic acid, apigenin, luteolin, *p*-coumaric acid, and chlorogenic acid [65]. In the present study, we found weak antioxidant activity, which could be the result of the poor solubility of polyphenols in the water extract [66]. For example, the *S. aethiopis* water extract and *S. microstegia* ethanol extract presented an activity similar to ascorbic acid, but the lowest activity was observed in the *S. microstegia* water extract [65].

Quercetin-7-*O*-diglucoside isolated from stem bark and wood extracts of *Terminalia brownii* possessed significant antifungal activity against *Aspergillus* and *Fusarium* strains [67]. In addition, quercetin and its derivatives have been reported to have good antifungal activities with low MIC values [68,69,70,71]. The growth of *Fusarium* spp. has been suppressed by dihydroquercetin isolated from barley [72]. Naringenin and its derivatives demonstrated some antifungal and antibacterial activity [73].

It should be mentioned that, while the flavonoid compounds relies on the comparison of their retention times with standard compounds, there still the biggest peaks in the chromatograms at 2.97 and 5.35 min which could be contributed properly the assessed biological activities, especially the identified flavonoids seem to be minor components in the water extract of *A. saligna*. These results supported the limitations of the HPLC analysis when using the available standard compounds.

Overall, it can be concluded from the current study that the water extract from flowers, containing various phenolic compounds, can be used as a bio-fungicide against certain wood-staining molds.

## 4. Materials and Methods

### 4.1. Plant Material and Preparation of the Extract

Flowers of *Acacia saligna* were collected from Alexandria, Egypt. The extraction procedure was carried out according to Salem et al. [74], with some modification, where approximately 25 g of the flowers were extracted with distilled water (200 mL) for 3 h under heat using a water bath at 50 °C. The extract was filtered with cotton plugs and then with filter paper (Whatman No. 1) (Mumbai, India) and concentrated to a small volume using a rotary evaporator (Rotavapor RII, Cole-Parmer GmbH, BucHI, Essen Germany). The water extract (7.45% *w/w* of fresh weight) was stored in a brown vial prior to chemical and bioactivity analyses.

### 4.2. Fungal Isolation, DNA Extraction, PCR, and Sequencing

During 2017, the tissues of infected *Citrus sinensis* L. trees showing root rots, cankers, and green fruit rot symptoms (Beheira, Egypt) were collected from Beheira governorate in Egypt and sent to the Plant Pathology laboratory of the Agricultural Botany Department, Alexandria University, Alexandria, Egypt, for isolation of the causal agents. The fungal isolates were isolated, kept on potato dextrose agar (PDA) plates, and incubated for seven days. The cultural and morphological characteristics of the isolated fungi were used for identification to the genus level, and for further molecular characterization, the DNA extraction was carried out on freshly growing mycelium using the GenElute™ Plant Genomic DNA Miniprep Kit (Sigma-Aldrich, St. Louis, MO, USA) following the manufacturer’s instructions. The PCR was carried out using primer pairs (ITS1/ITS4) to amplify the internal transcribed spacer (ITS) region of the rDNA [75,76].

The PCR reaction mixture consisted of a total volume of 25 µL; made up of 1 µL target DNA, 2.5 µL of 10 × PCR buffer (Sigma-Aldrich, St. Louis, MO, USA), and 1.25 µL deoxynucleotide triphosphates (dNTPs) mix (2.5 mM each); 1 µL each of the sense and antisense primer; 1 µL MgCl_2_ (50 mM); 0.1 µL Taq polymerase (Sigma-Aldrich, St. Louis, MO, USA); and RNAse free water, which was used to reach a total of 25 µL.

The reaction was carried out in a Techne Prime thermal cycler (Techne, Cambridge, United Kingdom). The reaction cycle consisted of denaturation for 4 min at 95 °C; followed by 40 cycles of 1 min at 94 °C, 1 min at 50 °C, and 1 min at 72 °C; and a final extension of 8 min at 72 °C. Amplification products were separated in 1.2% (*w/v*) agarose gel (iNtRON Biotechnology, Inc., Seongnam, South Korea), and pre-stained with red safe (iNtRON Biotechnology, Inc., Seongnam, South Korea) along with a 100 bp plus ladder at 70 V. The DNA bands were observed under a UV transilluminator. Purified fragments of ITS were sequenced by Macrogen, Inc., Seoul, Korea. The generated sequences were deposited in GenBank under the accession numbers presented in Table 1.

### 4.3. Antifungal Activity of Wood Treated with Water Extract

**Figure 5:**
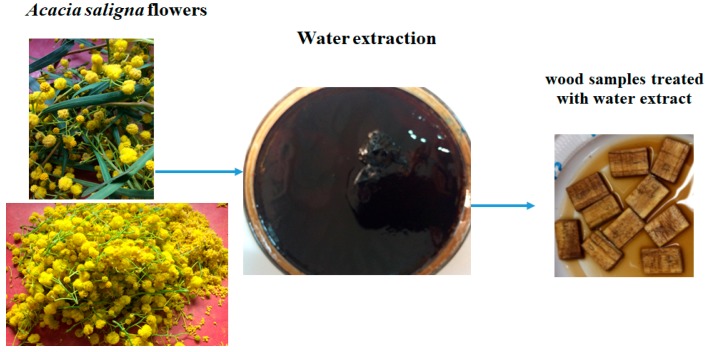
Wood samples treated with water extract of *A. saligna* flowers

The water extract from *A. saligna* flowers was prepared at concentrations of 0%, 1%, 2%, and 3% by dissolving the extract in 10% dimethyl sulfoxide (DMSO). A total of 36 wood samples of *Melia azedarach* with dimensions of 0.5 cm × 1 cm × 2 cm, air-dried, and autoclaved at 121 °C for 20 min, were used for the antifungal activity test (Figure 5). Three wood samples were used for each concentration. The antifungal activity was evaluated against the linear growths of *Fusarium culmorum*, *Rhizoctonia solani*, and *Penicillium chrysogenum*. The inhibition percentage of mycelial growth was calculated using the following equation: Mycelial growth inhibition (%) = [(A_C_ − A_T_)/A_C_] × 100 [77], where A_C_ and A_T_ are average diameters of the fungal colony of the control and treatment, respectively. Wood samples soaked only with 10% DMSO were used as the control.

### 4.4. Antibacterial Activity

The minimum inhibitory concentrations (MICs) of water extracts from *A. saligna* flowers were evaluated against the four phytopathogenic bacteria *Agrobacterium tumefaciens*, *Enterobacter cloacae*, *Erwinia amylovora*, and *Pectobacterium carotovorum subsp. carotovorum* using the micro-dilution method with serial concentrations of 4–350 µg/mL [78], and compared with the positive control (Tobramycin 10 μg/disc).

### 4.5. Determination of Antioxidant Activity

The antioxidant capacity was assessed by the 2,2′-diphenylpicrylhydrazyl (DPPH) assay [79] in terms of IC_50_ (the concentration that caused a 50% inhibition of growth compared with control) using the calibration curve compared with butylated hydroxytoluene (BHT).

### 4.6. HPLC condition for Phenolic Compounds

An Agilent 1260 Infinity HPLC Series (Agilent, Santa Clara, CA, USA), equipped with a Quaternary pump and a Zorbax Eclipse plus C18 column (100 mm × 4.6 mm i.d.) (Agilent Technologies, Santa Clara, CA, USA), was operated at 30 °C. Separation was achieved using a ternary linear elution gradient with (A) HPLC grade water 0.2% H_3_PO_4_ (*v/v*), (B) methanol, and (C) acetonitrile. The injected volume was 20 μL. A VWD detector was set at 284 nm. The standard phenolic compounds used were gallic acid, catechol, *p*-hydroxy benzoic acid, caffeine, vanillic acid, caffeic acid, syringic acid, vanillin, *p*-coumaric acid, ferulic acid, ellagic acid, benzoic acid, *o*-coumaric acid, salicylic acid, and cinnamic acid.

### 4.7. HPLC Condition for Flavonoids

HPLC, Smart line, Knauer, Germany, equipped with a binary pump, a Zorbax Eclipse plusC18 (column 150 mm × 4.6 mm i.d.) (Agilent technologies, USA), operated at 35 °C, was used. The conditions used were: Eluent as methanol: H_2_O with 0.5% H_3_PO_4_, 50:50; a flow rate of 0.7 mL/min; and injected volume of 20 μL. The UV detector was set at 273 nm and data integration was done using ClarityChrom@ Version 7.2.0, Chromatography Software (Knauer Wissenschaftliche Geräte GmbH, Hegauer Weg 38, 14163 Berlin, Germany). The standard flavonoid compounds were rutin, myricetin, quercetin, naringenin, kaempferol, and apigenin.

### 4.8. Statistical Analysis

The results of the inhibition percentage of mycelial growth of the three fungi as affected by the four concentrations (0, 1, 2, and 3%) of the *A. saligna* flower water extract were statistically analyzed using one-way analysis of variance (ANOVA) SAS software (SAS Institute, Release 8.02, Cary, North Carolina State University, Raleigh, NC, USA), and the means were compared against the control treatment.

## 5. Conclusions

In the present study, the water extract of *A. saligna* flowers at 3% shows moderate antifungal properties against the three mold species tested (*F. culmorum*, *R. solani*, and *P. chrysogenum*). Therefore, it is possible to assert that there are some potential applications of this extract for wood protection. The water extract showed lower antibacterial and antioxidant activities than the standards used, Tobramycin and butylated hydroxytoluene (BHT), respectively. Overall, the presence of the activity of the water extract could be related to the presence of some phenolic and flavonoid compounds.

## Figures and Tables

**Table 1:** Accession numbers of fungal isolates used for antifungal activity evaluation.

Fungal Isolate	Accession Number
*Fusarium culmorum*	MH352452
*Rhizoctonia solani*	MH352450
*Penicillium chrysogenum*	MH352451

**Table 2:** Mycelia percentage inhibited of *F. culmorum*, *P. chrysogenum*, and *R. solani* by wood treated with *A. saligna* flower water extracts at different concentrations.

Conc. (%)	Inhibition Percentage of Mycelial Growth (%)		
	*F. culmorum*	*P. chrysogenum*	*R. solani*
	Mean ± SD	Mean ± SD	Mean ± SD
0	0.00 ^c^	0.00 ^c^	0.00 ^b^
1	31.11 ^b^ ± 2.22	14.07 ^c^ ± 7.14	40.74 ^a^ ± 1.28
2	31.11 ^b^ ± 2.22	36.29 ^b^ ± 1.28	41.48 ^a^ ± 1.28
3	38.51 ^a^ ± 1.28	65.92 ^a^ ± 1.28	41.48 ^a^ ± 1.28
*p*-value	<0.0001	<0.0001	<0.0001
LSD_0.05_	3.195	6.938	2.092

Conc. = Concentration. Means with the same superscript letters within the same column are not significantly different according to LSD_0.05_.

**Table 3:** The MIC (µg/mL) values against the growth of four phytopathogenic bacteria.

Tested Material	MIC (µg/mL)			
	*A. tumefaciens*	*E. cloacae*	*E. amylovora*	*P. carotovorum subsp. carotovorum*
Extract	200	300	300	100
Tobramycin (10 μg/disc)	32	35	35	16

**Table 4:** Chemical composition analysis of phenolic and flavonoid compounds of water extract from *A. saligna* flowers by HPLC.

Compound	Conc. (mg/100 g)
Phenolic compounds	
Gallic acid	ND *
Catechol	6.54
*p*-Hydroxy benzoic acid	14.13
Caffeine	100.11
Vanillic acid	ND
Caffeic acid	2.50
Syringic acid	5.83
Vanillin	ND
*p*-Coumaric acid	2.45
Ferulic acid	6.65
Ellagic acid	12.17
Benzoic acid	161.68
*o*-Coumaric acid	42.09
Salicylic acid	4.43
Cinnamic acid	ND
Flavonoid compounds	
Rutin	ND
Myricetin	ND
Quercetin	111.96
Naringenin	145.03
Kaempferol	44.49
Apigenin	ND

* ND: not detected.
